# Association between secondhand smoke exposure at home and cognitive performance among rural primary school children in Malaysia

**DOI:** 10.18332/tid/133638

**Published:** 2021-04-15

**Authors:** Zulkarnain Ellis-Suriani, Bachok Norsa’adah, Azizah Othman, Ab Hamid Siti-Azrin

**Affiliations:** 1 Unit of Biostatistics and Research Methodology, School of Medical Sciences, Universiti Sains Malaysia, Kota Bharu, Malaysia; 2 Department of Pediatrics, School of Medical Sciences, Universiti Sains Malaysia, Kota Bharu, Malaysia

**Keywords:** smoking, secondhand smoke, cognitive function, school children, rural

## Abstract

**INTRODUCTION:**

Secondhand smoke (SHS) exposure is linked to a series of serious health problems. Children may be particularly vulnerable to the effects of SHS exposure at home. This study aimed to determine the association between SHS exposure at home and cognitive performance in school children.

**METHODS:**

A multistage sampling was performed across rural primary schools in Kuala Krai, Kelantan, Malaysia. Data were collected using self-administered questionnaires and the children aged 10–11 years (n=312) were subjected to cognitive tests including digit span, letter-number sequencing, coding, and symbol search. Cognitive performance was tested using subscales derived from the Wechsler Intelligence Scale for Children.

**RESULTS:**

The prevalence of SHS exposure at home was 55.8%, where 11.9% of children lived with one smoker, while 43.9% of children lived with ≥2 smokers. There was a significant difference in the mean score of the combined cognitive tests between SHS-exposed and non-exposed children after adjustment for sex, parental educational level, family income and academic performance [Pillai’s Trace=0.084, F statistic (df)=6.803 (4302), p<0.001].

**CONCLUSIONS:**

More than half of the primary school children in rural Kuala Krai were exposed to SHS from at least one smoker at home. There was a significant association between SHS exposure at home and cognitive performance.

## INTRODUCTION

Smoking is the cause of more than seven million deaths per year worldwide^1^. More than 85% of those fatalities are the result of direct smoking, whereas approximately 0.89 milion are due to secondhand smoke (SHS) exposure^1^. Malaysia is a developing country with a high smoking prevalence (21.1%) compared with for instance Cambodia (14.8%), Singapore (16.1%), Brunei (16.9%), and Japan (20.6%)^1^. According to the National Health and Morbidity Survey, the overall prevalence of current smokers aged ≥15 years was 22.8% in 2015, reflecting 43% of the males and 1.4% of the females^2^. In 2015, the percentage of male smokers had decreased by 0.9%, whereas an increase of 0.4% was observed among female smokers^2^. Almost a quarter of Malaysian adults are smokers, and thus the exposure to SHS in the general population is suspected to be substantial. The Global Adult Tobacco Survey 2011 of Malaysia reported that approximately 27.9% of non-smoking adults were exposed to SHS at home^3^. The National Health and Morbidity Survey 2019 reported that 31% of Malaysians were exposed to SHS at home, compared with 27% at work^4^. A Morbidity and Mortality Weekly Report of US reported that 25.2% of non-smokers aged ≥3 years had serum cotinine levels of 0.05–10 ng/mL^5^.

The prevalence of SHS in Malaysia is higher in the rural than in the urban population, and this trend has been consistent over the years. The National Health and Morbidity Survey in 2019 reported a prevalence of SHS at home of 40.3% and 28.3% in rural and urban areas, respectively^4^. These figures were lower than those reported in 2015, where 33.3% of the urban population had been exposed to tobacco smoke at home, as opposed to 48.8% in the rural population^2^. The difference between urban and rural areas in the prevalence of SHS is related to: 1) sociodemographic factors of rural areas, such as lower income and education levels and higher levels of unemployment; 2) tobacco control policies and other regulatory factors, which benefit urban areas more than rural areas; 3) low population density in rural areas; 4) limited health services and information on smoking in rural areas; and 5) the fact that tobacco crops represent a major commodity in some rural areas^6^.

Malaysia is one of the countries that acceded to the WHO Framework Convention on Tobacco Control in 2005^7^. Smoking is prohibited in specific public places and at workplaces. Individuals aged <18 years are prohibited from smoking, and any form of tobacco advertising and promotion are banned. However, people in Malaysia are less compliant to this smoke-free legislation. Active smokers can be found in restricted premises, which contain significantly higher PM2.5 concentrations than other premises without active smokers^8^.

SHS is smoke from the combustion of tobacco products, such as cigarettes, cigars or pipes. SHS is also referred to as passive smoking, environmental tobacco smoke or involuntary smoking, where it is a mixture of side-stream and mainstream smoke. Side-stream smoke is the smoke coming from the burning tip of a cigarette or other smoked tobacco product, while mainstream smoke is the smoke exhaled by a smoker that is diluted by the surrounding air^9,10^. SHS is a major preventable cause of morbidity and mortality in adults and children^11^. The smoke is harmful to both children and adults, and the only way to protect non-smokers is to eliminate smoking at home, in worksites and in other enclosed places^11^. Exposure to SHS increases the risk of several diseases, such as heart disease, lung cancer, bronchitis and sudden infant death syndrome. Children exposed to this environmental hazard are at particular risk of developing adverse health outcomes, and may experience impaired respiratory development in particular^12^.

Exposure to SHS is not only associated with a range of health-related problems, it is also linked to adverse effects on cognitive performance^9^. Cognitive performance is the ability to mentally process received information, use and manipulate it whenever necessary, and apply reasoning to the information. The complex functions of cognition include language skills, perception, learning, attention, memory and decision-making. Cognitive functions can be measured using various methods; however, the gold standard is objective testing using a standardized, psychometrically sound instrument. This measurement includes a neuropsychological battery of test or subtests from a single battery. Studies have shown that exposure to SHS during childhood may impair neurodevelopmental processes^12^. Nicotine may affect the area of the brain involved in attention, memory, and learning. Cognitive abilities such as reading and calculating were reduced among children aged 6–16 years when exposed to SHS^13^. A study showed that children exposed to household smoking had altered cognitive function and reduced academic capabilities^14^.

Meanwhile, there is a lack of studies on the association between SHS exposure and cognitive function among children in Malaysia, particularly in rural areas. The present study aimed to determine the prevalence of SHS among children in the rural area of Kuala Krai and its association with cognitive function. The results of this study could be used to inform campaigns on health hazards associated with both active and passive smoking.

## METHODS

We used a cross-sectional study design. We selected school children using the multistage sampling method. Of the 34 schools in the rural area of Kuala Krai Kelantan, four primary schools were selected. In each school, we listed all the eligible children and randomly selected 78. Overall, we included 312 children. These schools and children were randomly selected using a random table. In this study, we defined children as those who were aged 10–11 years. We selected only students from the fourth and fifth grades and their parents who were able to understand and read Malay. Children with learning disabilities as reported by their teachers and those who had a sibling already enrolled in the study were excluded.

The sample size was calculated using the two means formula^15^. With 95% confidence level, 80% power, 1.77 standard deviation of the mean of digit span score^16^, a ratio of 1 and a design effect of 2, the required sample size was calculated as 312, including 10% drop out samples.

The study outcome was cognitive performance, which was operationally defined as the scores on four selected subscales derived from the Wechsler Intelligence Scale for Children (WISC-V)^17^, a set of tests designed to measure the intelligence and cognitive ability of children aged 6–16 years. This study included only nonverbal performance tests, including digit span, letter-number sequencing and coding, and symbol search subtests. The tasks in each subtest were carefully designed to measure the individuals’ current, complex cognitive abilities, particularly their attention, concentration, memory, speed and accuracy of visual identification, as well as decision-making and implementation skills. The tests were individually administered to the children by a single highly trained assessor who followed a fixed procedure and was unaware of the children’s status to SHS exposure.

In this study, the independent factor was exposure to SHS. Respondents were classified into SHS-exposed and non-exposed children. Home exposure to SHS was defined as the exposure of a child to tobacco combustion products due to smoking by the parent(s) or other people who smoked at least once in the past week inside the house in the presence of the child^4,16,18,19^. A non-exposed child was defined as one who was not exposed to SHS at home. Information on sociodemographic and smoking status of the parents or guardian(s) was collected using a self-administered questionnaire^16^. All school children were given a set of questionnaires to be given to their parents or guardian(s) to be completed. This study was performed in accordance with the Declaration of Helsinki. Ethical approval for this study was obtained from the Human Research Ethics Committee, University Sains Malaysia: USM/JEPeM/19070384. Permission was also obtained from the Ministry of Education, Malaysia and Kuala Krai District Education Office.

### Statistical analysis

Data entry and data analysis were performed using the Statistical Package for Social Science version 24.0. Means, standard deviations (SD), medians, interquartile ranges (IQR), frequencies and percentages were presented for the descriptive analysis. Multivariate Analysis of Covariance (MANCOVA) was used to determine the mean differences in digit span, letter-number sequencing, coding and symbol search in combination between SHS-exposed and non-exposed children with adjustment for covariates and confounders. A p<0.05 was considered statistically significant.

## RESULTS

A total of 312 respondents were included in the study, 53.5% were females and 92.6% were of Malay ethnicity. A total of 63.1% of the fathers and 42.9% of the mothers had received secondary education (Table 1). The median family income was MYR 800 (IQR: 1300) (MYR: 100 Malaysian Ringgits about US$24). We found that 52.6% of the respondents lived in wooden houses, whereas 47.4% lived in brick houses. The median number of rooms and windows in the houses were three (IQR: 1) and seven (IQR: 3), respectively.

**Table 1:** Sociodemographic characteristics and home environment of rural primary school children in Kuala Krai (N=312)

*Characteristics*	*n (%)*
**Gender**	
Male	145 (46.5)
Female	167 (53.5)
**Ethnicity**	
Malay	289 (92.6)
Non-Malay	23 (7.4)
**Education level of father**	
No formal	16 (5.1)
Primary	65 (20.9)
Secondary	197 (63.1)
Tertiary	34 (10.9)
**Education level of mother**	
No formal	43 (13.8)
Primary	80 (25.6)
Secondary	134 (42.9)
Tertiary	55 (17.6)
**Family income** (MYR)^a^	800.00 (1300.00)^b^
**Type of house**	
Wooden	164 (52.6)
Brick	148 (47.4)
Rooms in house^a^	3.00 (1.00)^b^
Windows in house^a^	7.00 (3.00)^b^

a Positive skewness.

b Median (IQR). IQR: interquartile range.

MYR: 100 Malaysian Ringgits about US$24.

The percentage of exposure to SHS at home by at least one smoker was 55.8% (95% CI: 50.3–61.2). There were 37 (11.9%) respondents living with one smoker and 137 (43.9%) with two or more smokers. Table 2 shows that 61.2% of the fathers and 45.8% of others, including brothers and uncles, who were living with the children were smokers. In total, 88.5% of the fathers, and all brothers and uncles who smoked did so inside the houses. None of the mothers was a smoker. The median number of cigarettes smoked per week among the fathers and others were 70 (IQR: 119) and 40 (IQR: 15), respectively. The most common tobacco products used among the smokers were cigarettes, which were smoked by 83.8% of the fathers and 85.3% of the brothers and uncles.

**Table 2:** Smoking status of parents or other smokers among rural primary school children in Kuala Krai (N=312)

*Variables*	*n (%)*
**Smoking status**	
**Father**	
Yes	191 (61.2)
No	121 (38.8)
**Others**	
Yes	143 (45.8)
No	169 (54.2)
**Exposed children**	
One smoker	37 (11.9)
Two or more smokers	137 (43.9)
Non-exposed children	138 (44.2)
***Father*** (n=191)	
**Smoking at home**	
Yes	169 (88.5)
No	22 (11.5)
Cigarettes smoked at home/week^a^	70.00 (119.00)^b^
**Type of tobacco product used**	
Cigarette	160 (83.8)
Cigar	15 (7.9)
Rolled tobacco	16 (8.3)
***Others*** (n=143)	
Uncle	78 (54.0)
Brother	65 (45.0)
**Smoking at home**	
Yes	143 (100.0)
No	0
Cigarettes smoked at home/week^a^	40.00 (15.00)^b^
**Type of tobacco product used**	
Cigarette	122 (85.3)
Cigar	17 (11.9)
Rolled tobacco	4 (2.8)

a Positive skewness.

b Median (IQR).

IQR: interquartile range.

The mean scores of each of the cognitive function tests were compared (Table 3). All the tests showed higher scores in the non-exposed group compared with the SHS-exposed group. However, a significant mean difference between SHS-exposed and non-exposed children was observed only for the digit span test, and no significant difference in mean scores was observed for each of the letter-number sequencing, coding or symbol search tests.

**Table 3:** Comparisons of mean score of cognitive tests between exposed and non-exposed children to SHS (N=312)

*Cognitive tests*	*Mean (SD)*		*Mean difference (95% CI)*	*t statistic (df)*	*p ^a^*
	*Exposed n=174*	*Non-exposed n=138*			
**Digit span**	11.20 (3.50)	13.75 (3.93)	2.55 (1.63–3.46)	5.45 (310)	0.012
**Letter–number sequencing**	11.09 (5.38)	11.42 (5.44)	0.33 (-0.76–1.42)	0.59 (310)	0.555
**Coding**	40.56 (10.40)	41.36 (10.26)	0.79 (-1.53–3.11)	0.67 (310)	0.502
**Symbol search**	24.70 (5.73)	25.17 (6.96)	0.47 (-0.94–1.88)	0.65 (310)	0.513

a Independent t-test.

Nevertheless, a significant mean difference of cognitive functions in combination (digit span, letter-number sequencing, coding and symbol search) between SHS-exposed and non-exposed children was found when adjustments were made for sex, parental educational levels, family income and academic performance [Table 4; multifactorial MANCOVA with Pillai’s Trace=0.08; F statistic (df)=6.80 (4302); p<0.001]. When the dependent variables were considered separately using multifactorial ANCOVA, a significant difference in the mean scores of the digit span test (p<0.001) was revealed after Bonferroni correction. The non-exposed children achieved significantly higher scores in the digit span test with adjusted mean 13.54 (95% CI: 12.66–14.42) than SHS-exposed children with adjusted mean 11.10 (95% CI: 10.30–11.91) with p=0.010. Other tests did not have any significant difference in the mean scores between SHS-exposed and non-exposed children.

**Table 4:** Association between SHS exposure and cognitive performance test (digit span, letter–number sequencing, coding and symbol search) (N=312)

*Cognitive tests*	*Group*	*n*	*Adjusted mean (95% CI)*	*F statistic (df)*	*p ^a^*
**Digit span**	Exposed	174	11.10 (10.30–11.91)	26.54 (1305)	0.010
	Non-exposed	138	13.54 (12.66–14.42)		
**Letter–number sequencing**	Exposed	174	10.20 (9.26–11.15)	0.66 (1305)	0.422
	Non-exposed	138	10.65 (9.62–11.69)		
**Coding**	Exposed	174	40.22 (38.16–42.29)	0.52 (1305)	0.468
	Non-exposed	138	41.09 (38.84–43.34)		
**Symbol search**	Exposed	174	24.83 (23.58–26.07)	0.46 (1305)	0.503
	Non-exposed	138	25.32 (23.96–26.69)		

a Test of between subject effects.

Multifactorial MANCOVA Pillai’s Trace=0.084, F statistic (df)=6.80 (4302), p<0.001; with adjustment for gender, parents’ educational levels, family income and academic performance. Assumption of multivariate normal distribution, homogeneity of variance and covariance, multicollinearity, linearity between study factor and covariate were all fulfilled.

## DISCUSSION

This study revealed that the prevalence of SHS exposure at home among schoolchildren aged 10– 11 years in Kuala Krai was 55.8%. This result was based on the definition of having at least one family member smoking at home in the past week^4,16,18^. The prevalence of SHS exposure was higher compared with other studies, regardless of rural or urban area. The National Health and Nutrition Examination Survey, United States 2013–2014 reported that 37.9% of non-smokers aged 3–11 years had serum cotinine levels in the range of 0.05–10 ng/mL indicating exposure to SHS^5^. This age group had the highest percentage of serum cotinine levels compared with children in other age groups (32%) or adults (22%). Similarly, a cross-sectional study among non-smoking schoolchildren aged 10–11 years in Malaysia reported that children living with smokers (either father or relatives) had significantly higher salivary cotinine concentrations than those living with non-smokers^19^. In addition, urban residences had a significantly positive association with high cotinine levels.

Most other studies on SHS involved older children or adolescents. A study by Ghazali et al.^9^ reported that 41.5% of adolescents included in the Global School Health Survey in Malaysia were exposed to SHS. A recent study reported that 46.8% of middle school students in Thailand had been exposed to SHS at home^20^. The National Youth Tobacco Survey reported that 44.5% of middle and high school students from the United States in 2014 were exposed to SHS through household smoking or electronic cigarettes^21^. In China, the prevalence of SHS exposure to adult smokers in urban and rural areas were 60.2% and 61.8%, respectively^22^. An Indian study by Singh and Sahoo^23^ reported SHS exposure rates at home of 34.5% and 53.7% in urban and rural areas, respectively. These data show that SHS exposure is generally higher in rural areas than in urban areas worldwide. This fact may be related to a low awareness among residents and the lack of smoke-free policies in rural areas^6^. Rural areas having higher smoking rates than urban areas, may also reflect demographic and psychosocial factors typically associated with rural areas, such as lower income and education levels and a higher rate of unemployment^14^. In addition, Doogan et al.^24^ found that tobacco control policies and other regulatory factors benefit urban areas more than rural areas. The low population density in rural areas may hamper communication efforts, including propagation of anti-smoking campaigns.

School children generally spend the majority of their time after school at home. Considering the amount of time spent at home, being exposed to SHS at home is a risk factor warranting public health awareness. Our study found that 61.2% of the fathers and 45.8% of other family members living with the children were smokers. In a study of schoolchildren aged 13–14 years in Thailand, the main source of SHS at home was smoking fathers (45.4%), relatives (24.1%), siblings (12.4%), mothers (3.3%), and neighbors and guests (14.8%)^20^. In a study on prenatal women in Malaysia, 94.9% had been exposed to SHS at home via their husbands but only 23.9% had been exposed to SHS from other housemates^18^. A study in rural Malaysia reported that 59.4% of primary school children had a father who smoked^25^. A study in Malaysia identified that 41.5% of adolescents aged 14 years had at least one smoking parent/guardian^9^.

There are a limited number of studies from Malaysia that focus of the effects of SHS on cognitive performance. The present study contributes data on the association between SHS exposure at home and cognitive performance among rural children. In line with this, a review of 15 articles revealed that SHS exposure is associated with poor neurocognitive performance among children in 12 articles^26^. Similarly, a study including children aged 6, 11 and 17 years and using the same instrument as our study found that SHS exposure was associated with a reduction in IQ Wechsler Intelligence Scale scores, but this association diminished after adjusting the maternal IQ and educational levels^27^.

Another Malaysian study conducted in rural– urban schools reported a non-significant association between SHS exposure and cognitive tests among children^16^, but that study did not adjust for possible confounders. Sharina et al.^16^, who adopted methods similar to our study, found that the scores of digit span, coding and arithmetic tests were not significantly different between the groups. A large study of children aged 6–16 years revealed significantly inverse relationships between serum cotinine and scores on reading, arithmetic and block design but not digit span^28^. In our study, only digit span had significantly higher scores in non-exposed than in exposed children. Notably, children who were not exposed to the SHS indeed scored consistently higher in all four cognitive tests. Both digit span and letter–number sequencing measure children’s auditory memory by requiring them to first pay adequate attention and concentrate and then verbally reproduce information they have memorized at the working memory level^29^. Digit span might pose an additional challenge, especially for children who have been exposed to the SHS, because the test taps into higher cognitive skills, such as manipulation of information in working memory and executive functioning. However, the reason why only the scores for digit span was significantly different remains unclear. Further exploration is needed.

### Strengths and limitations

Our study applied an observational design to reduce costs. It was based on an adequate and representative sample of a rural district with 100% response rate, which enables the generalization of the results to the rural children in Malaysia. We used a powerful multivariate statistical analysis controlling for possible confounders. This showed that the association between SHS exposure and cognitive function was independently related and un-affected by any confounders. Univariate and multivariate statistical analyses also showed similar results.

In this study, several measures of SHS exposure in children, recommended by Matt et al.^10^, were considered in the design of the study, including the following: 1) who uses tobacco (parents, relatives, neighbors, etc.); 2) where and when exposure takes place (home, car, bedroom, etc.); 3) contaminated media (air, carpets, toys, etc.); 4) how exposure takes place (inhalation, contact, etc.); 5) how much a child was exposed (biomarkers in urine, saliva, etc.); and 6) factors contributing to why tobacco is used in a child’s environment (community standard, culture, parental education, etc.). However, some of these measurements were not performed, and this is one of the study’s limitations. For instance, we did not measure the presence of particulate matter in ambient air of the children’s houses. Home survey and parent interview are the appropriate and accurate methods of collecting information about smoking exposures. However, in our study, children were selected from schools and were given questionnaires to be completed at home by their parents or guardian(s). This data collection method may underestimate the prevalence of SHS exposure. Furthermore, the exposure to SHS of this study was self-reported based on questionnaire, which can lead to measurement bias^24,26^. The exposure was not verified through biological measurement such as cotinine in the urine, saliva or hair of the children; thus, the exposure might be over- or under-estimated. In this study, we also relied on SHS exposure being constant over time in terms of the number of smokers who smoked at home on daily basis. There might be variation of dosage of exposure between the exposed children. We also did not investigate the frequency and duration of exposure and the vicinity of the children to the smokers. Such factors are prone to recall bias and thus not suitable for a cross-sectional study. We did not measure SHS exposure at other places, such as relatives’ homes and environments, which, however, would presumably minimal. We collected information on the type of house, number of rooms and windows in the house that may contribute to the amount of SHS exposure; however, these factors were non significantly associated with the study outcome. Further studies might consider including the other sources of SHS exposure to children. Finally, we adapted only four tests from the WISC-V for the cognitive performance test for the outcome of our study. There are five index scores in WISC-V, including the verbal comprehension, visual spatial, fluid reasoning, working memory and processing speed indices. Due to the time and logistic constraints, we measured only digit span and letter– number sequencing, which are part of the working memory index, and coding and symbol search, which are part of the processing speed index. We hope to include more tests in future studies.

## CONCLUSIONS

More than half of the rural school children studied were exposed to SHS at home, mainly via smoking fathers and other people living with the children. Home exposure to SHS was associated with lower cognitive performance. These results could be used as evidence of the health hazards associated with passive smoking among children. All parents should be aware of these effects to help prevent or stop SHS exposure at home, as the home is supposed to be a safe and conducive place for children.
